# Analysis of Gut Microbiota and Metabolites in Diannan Small Ear Sows at Diestrus and Metestrus

**DOI:** 10.3389/fmicb.2022.826881

**Published:** 2022-04-18

**Authors:** Xuancheng Guan, Junhong Zhu, Haichao Sun, Xiaoqi Zhao, Minghua Yang, Ying Huang, Hongbin Pan, Yanguang Zhao, Sumei Zhao

**Affiliations:** ^1^Yunnan Key Laboratory of Animal Nutrition and Feed Science, Yunnan Agricultural University, Kunming, China; ^2^Yunnan Province Key Laboratory for Porcine Gene Editing and Xenotransplantation, Yunnan Agricultural University, Kunming, China; ^3^Yunnan Academy of Animal Husbandry and Veterinary Sciences, Kunming, China; ^4^Shanghai Laboratory Animal Research Center, Shanghai, China

**Keywords:** Diannan small ear pig, sows, diestrus, metestrus, intestinal microbiota, 16S rRNA, LC-MS

## Abstract

The physiological state of the host affects the gut microbes. The estrus cycle is critical to the reproductive cycle of sows. However, the association between gut microbes and animal estrus is poorly understood. Here, high-throughput 16S rRNA sequencing and liquid chromatography-mass spectrometry (LC-MS) non-targeted metabolome technology were used to study the estrous cycles in Diannan small ear pigs. Significantly different gut microbiota and metabolites of sows at estrous and diestrus were screened out and the correlation was analyzed. We found that the intestinal microbial composition and microbial metabolism of Diannan small ear sows were significantly different at diestrus and metestrus. The abundances of *Spirochaetes*, *Spirochaetia, Spirochaetales*, *Spirochaetaceae*, *Deltaproteobacteria*, *unidentified_Alphaproteobacteria*, *Ruminococcus_sp_YE281*, and *Treponema_berlinense* in intestinal microorganisms of Diannan small ear sows at metestrus are significantly higher than that at diestrus. Propionic acid, benzyl butyrate, sucrose, piperidine, and 5-aminoimidazole-4-carboxamide ribonucleotide (AICAR) were significantly enriched at metestrus compared with diestrus, which were involved in the energy metabolism-related pathways and activated protein kinase (AMPK) signaling pathway. At diestrus and metestrus, differential microbiota of *unidentified_Alphaproteobacteria*, *Intestinimonas*, *Peptococcus*, *Terrisporobacter*, and differential metabolites of piperidine, propionic acid, and benzyl butyrate, sucrose, 4-methyl catechol, and AICAR exist a certain degree of correlation. Therefore, *unidentified_Alphaproteobacteria*, *Ruminococcus_sp_YE281*, and *Treponema_berlinense* may have a potential role at metestrus of the Diannan small ear sows. AICAR may be apotential marker of estrus Diannan small ear sows feces, but further studies about the specific mechanism are needed. These findings provide a new perspective for sows production management and improving sows reproductive performance.

## Introduction

The reproductive performance of sows directly affects the benefit of pig farms (Van Wettere et al., 2017). Estrus plays a vital role in the reproductive cycle of sows because effective estrus can promote reproduction performance (Andersson et al., 2016). A previous study established that Chinese indigenous pigs, such as Meishan, Erhualian, and Mi pigs, outperformed European pigs in terms of reproductive performance and estrous expression features, including larger litter sizes and a more distinct presentation of estrous behaviors (Bazer et al., 1988; Fan et al., 2002). Gilts of these Chinese pig breeds mature earlier. For instance, the mean age at puberty onset in Meishan gilts was 115 days, compared with 235 days in large white gilts (Hunter et al., 1993). They exhibit behavioral estrous for a little more extended time and have slightly shorter estrous cycles than landrace and large white (Bazer et al., 1988). Diannan small ear pig is a typical native pig species in China, with the above characteristics.

The gastrointestinal tract of mammals is composed of about 10^14^ microorganisms (Zhao et al., 2015; Yang et al., 2016). Intestinal microorganisms have a vast range of metabolic activities and produce abundant microbial metabolites, are often referred to as a “metabolic organ” of the host (LeBlanc et al., 2013). Intestinal microorganisms and their metabolites affect multiple physiological functions of the host, which can promote the absorption of nutrients and energy metabolism, promote healthy intestinal development, etc. (Backhed et al., 2004; Dai et al., 2010). The composition and abundance of intestinal microorganisms are directly related to the host animal’s health status and production performance. When the external environment, diet, and physiological state change, the composition and abundance of intestinal microorganisms will be impacted and then affect the host (Hang and Zhu, 2012).

Sex hormones affect the composition of the microbial community (Hooper et al., 2001; Velagapudi et al., 2010; Nicholson et al., 2012). On the contrary, the diversity of the microbial community also affects the metabolism of sex hormones. That is, the intestinal microorganisms and the host sex hormone secretion and metabolism have a two-way effect (Melvin, 2016). This study results suggest that there may be a specific correlation between intestinal microorganisms and animal estrus, and estrus, as an essential period in animal production, may also be closely related to intestinal microorganisms. However, there are still discrepancies between related studies conducted on various animal species. The results of a related study on Murrah buffalo and dholes suggest a reversible effect and a possible correlation between the gut microbiome and animal reproductive physiology (Wu et al., 2020; Sharma et al., 2021). On the contrary, relevant studies in female mice established that the estrous cycle does not result in any significant shift in the intestinal microbial community in the reproductively mature, regularly cycling female mouse (Guo et al., 2016; Wallace et al., 2018). As far as we are aware, no report exists on the intestinal microorganisms of estrus sows.

Therefore, this study aims to study the differences and correlations between intestinal microorganisms and metabolites in sows at diestrus and metestrus and provide a new perspective for sows production management and improvement of sows reproductive performance.

## Materials and Methods

### Animals

All experiment procedures were performed according to the Guide for Animal Care and Use of Laboratory Animals in the Institutional Animal Care and Use Committee of Yunnan Agricultural University. The Department Animal Ethics Committee approved the experimental protocol of the Yunnan Agricultural University. A total of six third-party Diannan small ear sows were chosen as the study object raised in the pig-breeding institute of the Yunnan Academy of Animal Husbandry and Veterinary Sciences. They were raised under a standardized feeding regimen with free access to water.

### Reproductive Cycle Determination and Fecal Collection

The feces of six Diannan small ear sows were collected and assigned to the DA group on the tenth day following the end of the sows’ previous estrus cycle, i.e., while the sows were in diestrus. Estrous detection was carefully performed twice ∼ three times daily at proestrus. The vulva’s reddening and swelling, as well as testing by a boar, were defined as standing reflexes of estrous expression and were scored visually using a standard scoring system (Rydhmer et al., 1994). The feces of six Diannan small ear sows were collected and assigned as the DC group when metestrus was detected in the sows.

Sterilized sampling tubes were quickly placed in a liquid nitrogen tank to collect the samples. Following their return to the laboratory, the feces samples were stored in a refrigerator set to −80°C.

### Genomic DNA Extraction and 16srRNA Gene Sequencing

The fecal DNA extraction kit was used to extract the total DNA of the samples (Beijing Tiangen Biochemical Technology Corporation Ltd.). The purity and concentration of DNA were determined by agarose gel electrophoresis and an appropriate amount of sample DNA was placed in a centrifuge tube. The sample was diluted to 1 ng/μl with sterile water. The diluted genomic DNA was used as the template for PCR expansion and the amplified region was the v3–v4 region of the 16S rRNA gene. The specific primers with barcode were used and the extended primers were 338F (5′-ACTCCTACGGGAGGCAGCA-3′) and 806R (5′-GGACTACHVGGGTWTCTAAT-3′). PCR products were detected by electrophoresis using 2% agarose gel. The PCR product was mixed with the same number of samples according to the concentration of the PCR product. After the samples were thoroughly mixed, the PCR product was purified by 1X TAE gel electrophoresis with a concentration of 2% and the target band was cut and recovered (Thermo Fisher Scientific Company GeneJET Adhesive Recovery Kit). Ion Plus Fragment Library Kit 48 RXNS from Thermo Fisher Scientific was used for library construction. After the library was qualified by Qubit quantitative and library testing, Ion S5™XL of Thermo Fisher Scientific was used for computer sequencing.

### Sequence Processing and Data Analysis

Cutadapt (V1.9.1)^1^ was used to cut low-quality parts of reading and then compared with the species annotation database to detect chimera sequences (Aßhauer et al., 2015). Chimera sequences were removed to obtain the final effective data (Clean Reads). All Clean Reads of all samples were clustered using Uparse software (Uparse v7.0.1001)^2^ (Haas et al., 2011), and by default, the sequence was clustered as operational taxonomic units (OTUs) with 97% identity. Species annotation of OTUs sequence was performed by Mothur method and SSUrRNA database of SILVA132 (the threshold was set as 0.8–1; Edgar, 2013). Fast multisequence alignment using the MUSCLE software (Version 3.8.31) to obtain system occurrence relationships for all the OTUs sequences (Quast and Pruesse, 2013). The samples were analyzed for alpha diversity and beta diversity by the QIIME software (Version 1.9.1). Unifrac distance was calculated by OTUs relation in samples (Lozupone and Knight, 2005; Lozupone et al., 2011), and Unweighted Pairgroup Method with Arithmetic Mean (UPGMA) were constructed. The LEfSe software was used for intergroup differential species analysis, and the screening value of linear discrimination criterion (LDA Score) was 2 (Segata et al., 2011). Use the Tax4Fun software to predict the function of all samples (Qin et al., 2012).

### Liquid Chromatography-Mass Spectrometry Analysis of Feca Metabolites

Take the liquid sample to be detected 100 μl (0.1 mg tissue liquid nitrogen grinding) placed in EP tube, add in 400 including 80% of methanol aqueous solution (four times the volume of methanol), vortex oscillation, in −20°C stand for 60 min, 14,000 *g*, 4°C centrifugal 20 min, take a certain amount of supernatant put in 1.5 ml centrifuge tube, vacuum freeze-drying, the residue with 100 μl complex solvents to dissolve, vortex oscillation, 14,000 *g*, 4°C centrifugal 15 min, take that into the supernatant fluid sample liquid chromatography-mass spectrometry (LC-MS) analysis.

Chromatographic conditions: positive model: mobile phase A: 0.1% formic acid, 95% acetonitrile, 10 mM ammonium acetate; mobile phase B: 0.1% formic acid, 50% acetonitrile, 10 mM ammonium acetate. Negative mode: mobile phase A: 95% acetonitrile, 10 mM ammonium acetate, pH 9.0; mobile phase B: 50% acetonitrile, 10 mM ammonium acetate, pH 9.0. Column temperature: 40°C; and flow rate: 0.3 ml/min. Mass spectrometry conditions: m/z 100–1,500 was selected for the scanning range. The settings of ESI are as follows: spray voltage: 3.2 kv; sheath gas flow rate: 35 arb; aux gas flow rate: 10 arb; and temperature: 320°C. Polarity:positive; Negative; MS/MS level 2 scans are data-dependent scans.

Import the output data (.raw) file into compound discoverer (CD) search software for simple filtering of retention time, mass charge ratio, and other parameters. Then, different samples were aligned according to the retention time deviation of 0.2 min and mass deviation of 5 PPM to make the identification more accurate. According to the set mass deviation of 5 PPM, signal strength deviation of 30%, the signal-to-noise ratio of 3, the minimum signal strength of 100,000, and other information, the peak was extracted. At the same time, the peak area was quantified, the target ions were integrated, the molecular formula was predicted and compared with the mz Cloud database, the background ions were removed with blank samples, the quantitative results were normalized with quality control (QC) samples, and finally, the identification and quantitative results of the data were obtained.

### Correlation Analysis

Pearson correlation analysis was conducted with SPSS version 22.0 and a visual relational network was constructed in Cytoscape software (v3.5.1) according to the correlation coefficient (*r*) of the different microbiota and metabolites.

## Results

### Gut Microbiota: Analysis and Function Prediction

**FIGURE 1:**
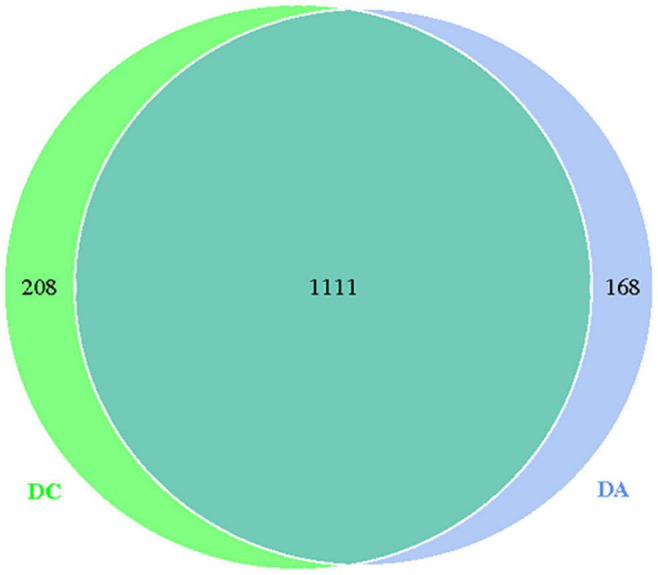
The OUT Wayne diagram.

Through the comparative analysis of OTUs of the two groups of samples, it was found that the total OTUs of the DA and DC groups were 1,111, while the OTUs of the DA and DC groups were 168 and 208, respectively (Figure 1).

**FIGURE 2:**
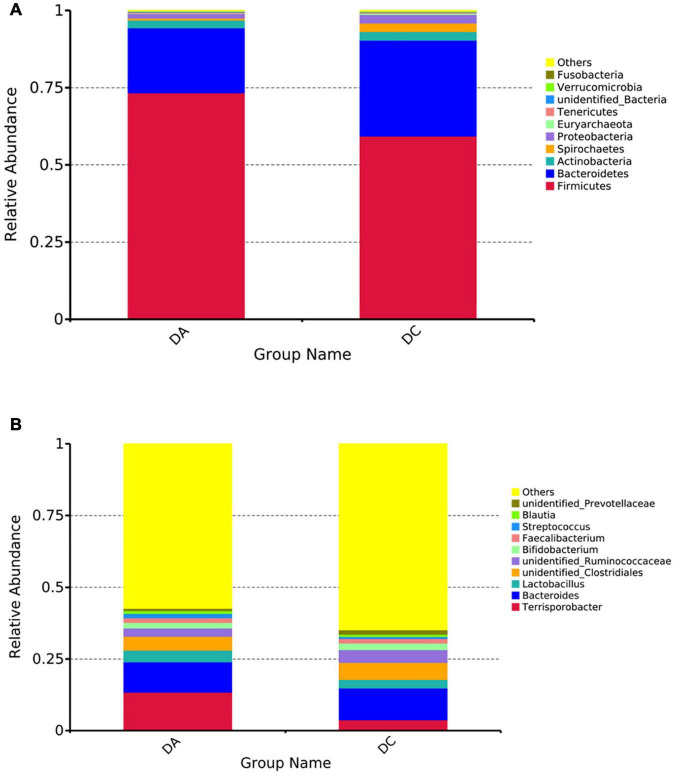
The histogram of relative abundance for intestinal microbiota in Diannan small ear sows (select the top 10 microbes at each level). **(A)** Phylum level, **(B)** genus level.

At the phylum level, a total of 24 phyla were noted, including *Firmicutes*, *Bacteroidetes*, *Actinobacteria*, *Proteobacteria*, *Spirochaetes*, etc., among which *Firmicutes* and Bacteroidetess were the major phyla of bacteria in both the DA and DC groups, accounting for more than 90% of total microbiota in both the groups (Figure 2A). At the genus level, a total of 218 genera were noted, including *Terrisporobacter*, *Bacteroides*, *unidentified_Clostridiales*, *Lactobacillus*, *unidentified_Ruminococcaceae*, etc. (Figure 2B).

**FIGURE 3:**
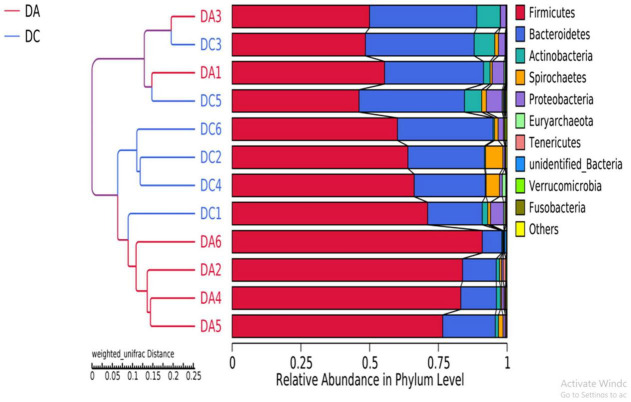
The beta diversity results of different grouped samples-UPGMA clustering tree.

After 97% identity clustering, the DA group got 801 OTUs on average and the DC group got 807 OTUs on average. OTUs are then further annotated to calculate and compare the alpha diversity among the samples (Table 1). However, there is no significant difference of Chao1, ACE, Shannon, Simpson, and PD whole-tree indexes between the DA and DC groups. The unweighted pair group method with weighted mean (UPGMA) showed that samples of the DA and DC groups are clustered in different branches, indicating that the microbiota structure of these two groups is different (Figure 3).

**TABLE 1:** The alpha diversity results of different grouped samples.

Sample name	DA	DC
Observed species	741 ± 83.62	748.5 ± 55.81
Shannon index	6.6 ± 0.89	6.97 ± 0.35
Simpson index	0.96 ± 0.04	0.98 ± 0.01
Chao1 index	804.64 ± 76.84	789.36 ± 59.16
ACE index	806.44 ± 76.92	796.25 ± 65.55
Goods coverage	0.998 ± 0	0.998 ± 0
PD whole tree	57.98 ± 5.85	59.26 ± 6.01

**FIGURE 4:**
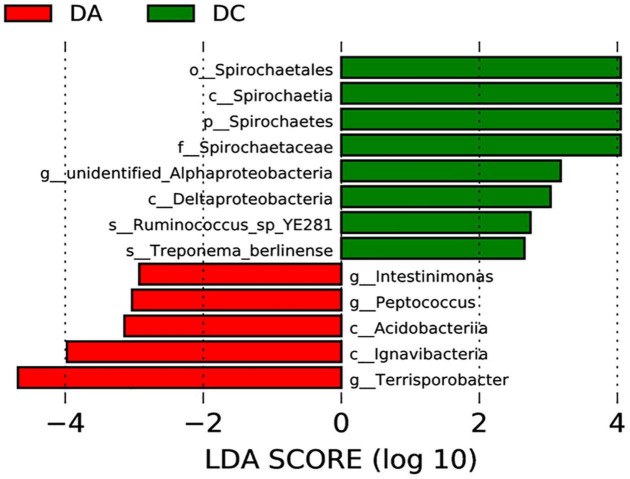
Analysis of LEfSe multilevel species difference discriminant.

The LEfSe multistage discriminant analysis showed that there were 13 biomarkers (LDA score = 2) in the intestinal microbial samples of pigs in the DA and DC groups (Figure 4). The relative abundance of *Spirochaetes* (phylum level), *Spirochaetia* and *Deltaproteobacteria* (class level), *Spirochaetales* (order level), *Spirochaetaceae* (family level), *unidentified_Alphaproteobacteria* (genus level), *Ruminococcus_sp_YE281*, and *Treponema_berlinense* (species level) in the DC group was significantly higher than that in the DA group (*P* < 0.05).

**FIGURE 5:**
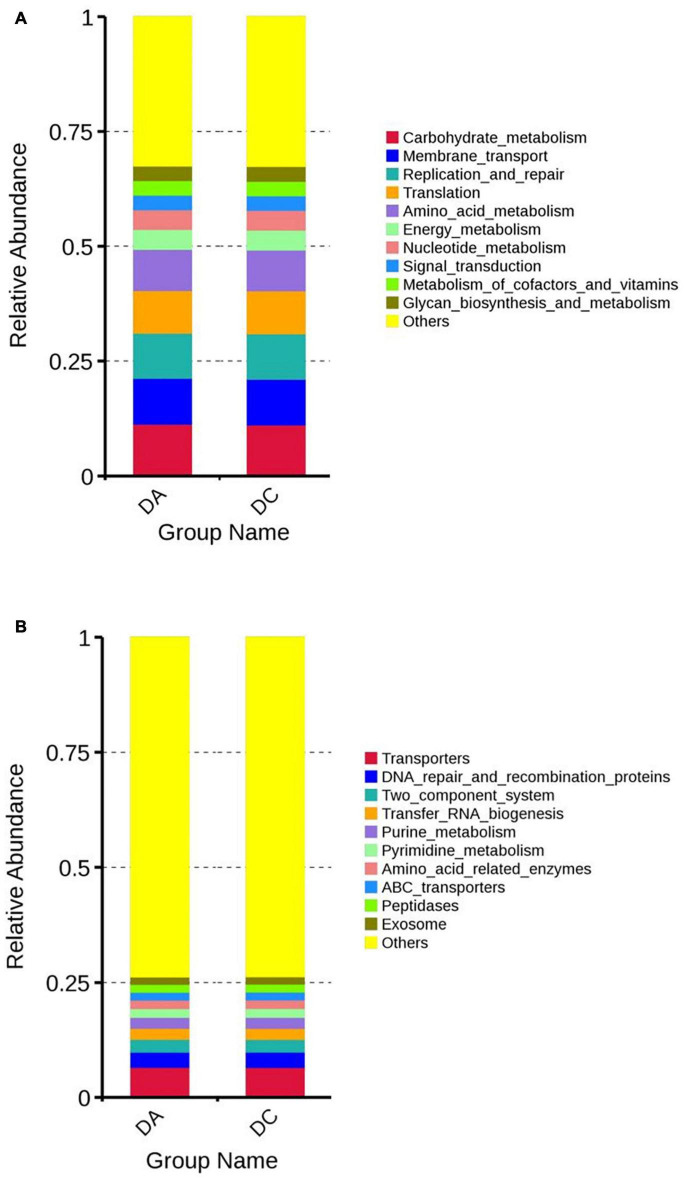
The function annotation abundance map of the KEGG level 2 **(A)** and level 3 **(B)**.

Tax4Fun software was used for functional prediction analysis. The results showed that at the Kyoto Encyclopedia of Genes and Genomes (KEGG) level 2, DA and DC samples were mainly involved in carbohydrate metabolism, transmembrane transport, replication and repair, translation, etc. At the KEGG level 3, DA and DC samples are mainly involved in transport proteins, DNA repair and recombinant proteins, two-component system, tRNA biosynthesis, etc. (Figure 5).

### Metabolome Data Analysis

**FIGURE 6:**
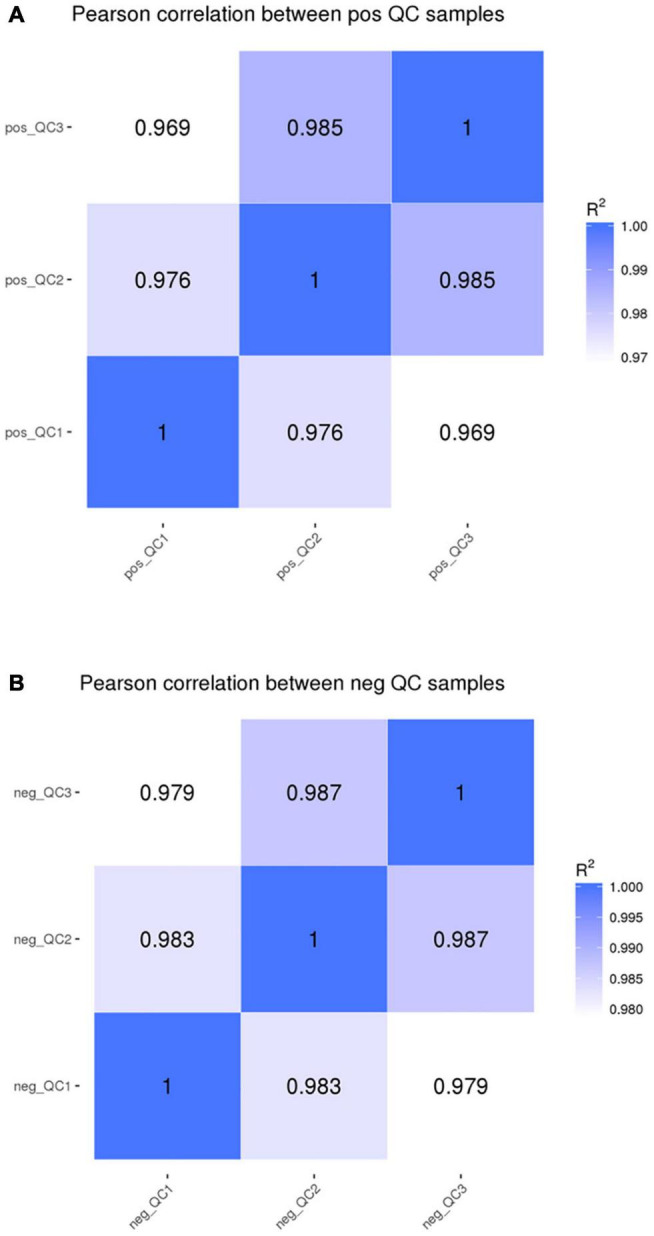
The quality control (QC) **(A)** chart positive ion mode **(B)** and negative ion mode.

**FIGURE 7:**
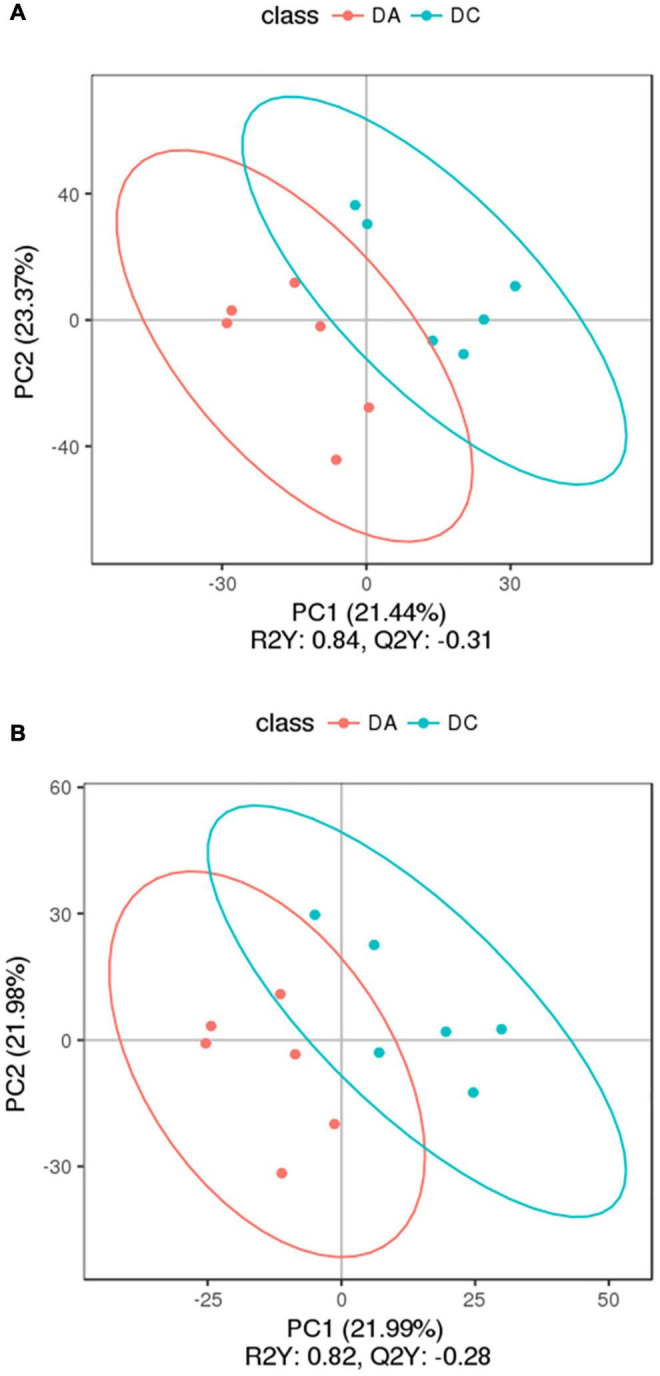
The partial least square-discriminant analysis (PLS-DA) chart. **(A)** Positive ion mode and **(B)** negative ion mode.

In the positive and negative ion modes, the correlation of QC samples (Figure 6) and R2 values are close to 1 (Figure 7). A total of 2,505 and 1,848 metabolites were detected in the fecal samples. It can be seen that oleic acid amide and pentadecanoic acid are the two metabolites with the highest relative expression under positive and negative ion mode, followed by roxatidine, myricoic acid, (+) -cp 55,940, oleic acid, etc.

**FIGURE 8:**
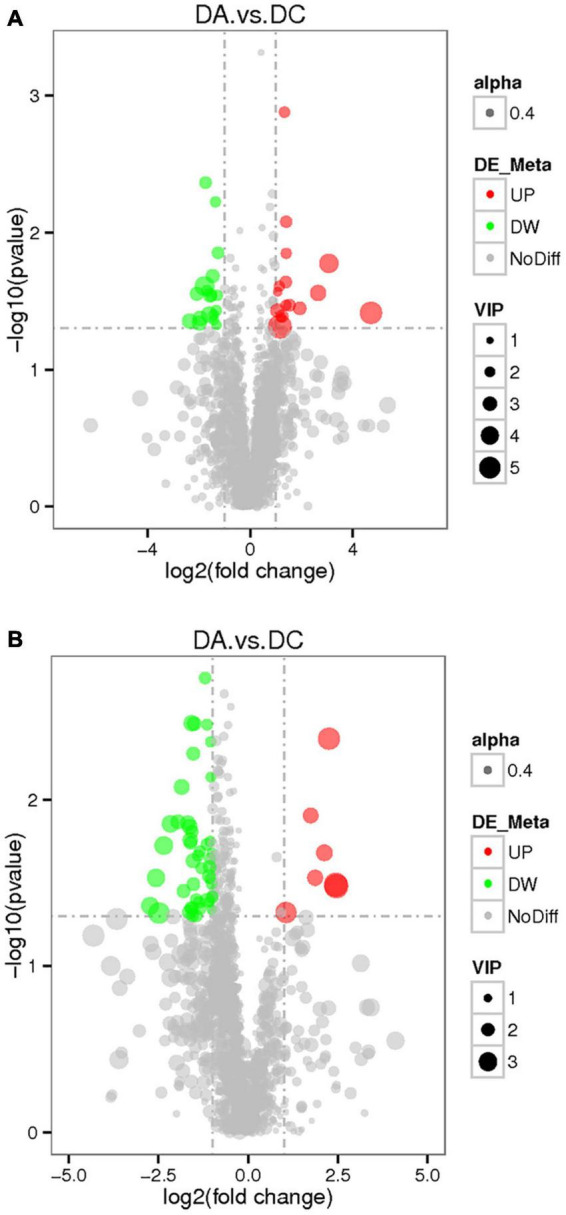
The volcano map of differential metabolites in estrus Diannan small ear sows. **(A)** Positive ion mode and **(B)** negative ion mode.

The threshold was set as VIP > 1.0, fold change FC > 2.0 or FC < 0.5 and *P* < 0.05, and the differentially expressed metabolites were screened out (Figure 8). In the positive ion mode, there were 34 metabolites with significant differences between the DC and DA groups, among which 18 metabolites were significantly upregulated and 16 metabolites were significantly downregulated. The differential metabolites were mainly dibenzothiophene, heophylline B, etc. There were 52 significantly different metabolites in the anion mode, of which 45 metabolites were significantly upregulated and 7 metabolites were significantly downregulated. The differential metabolites were mainly 6-hydroxy-1,2-adipate dioctyl ester, morpholinone, masalossiol, 2-ammonia-4-oxygen butyrate, etc.

**FIGURE 9:**
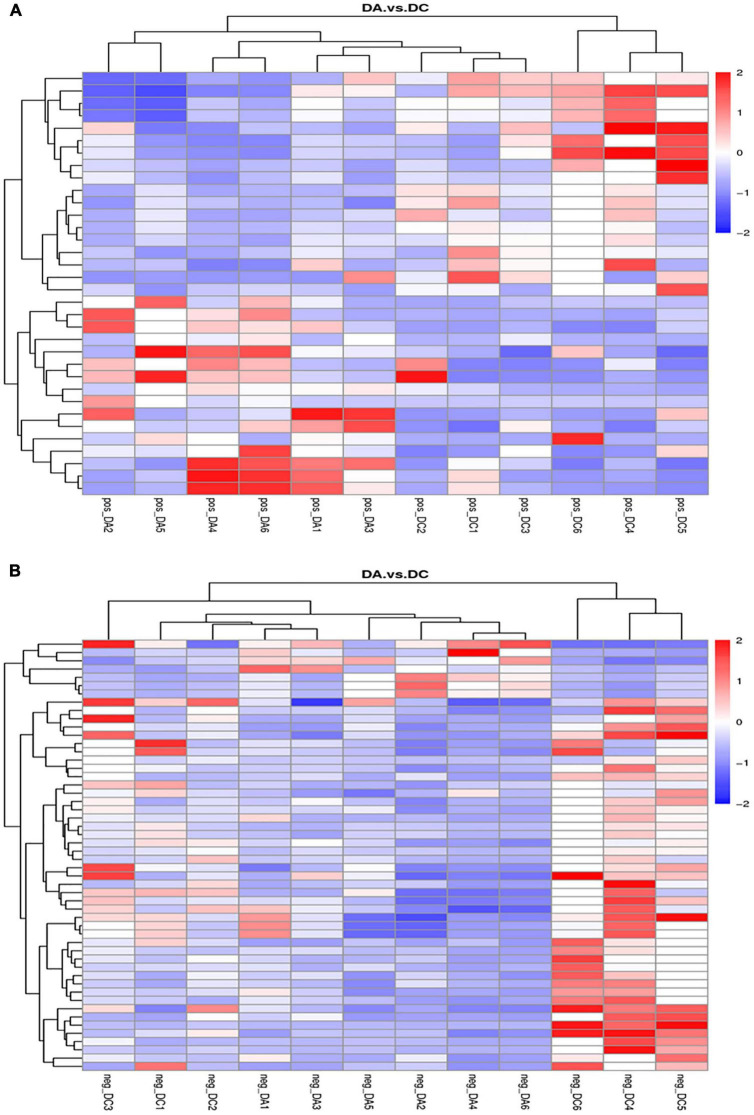
Clustering diagram of differential metabolites. **(A)** Positive ion mode and **(B)** negative ion mode.

The cluster analysis diagram showed that the differential metabolites in the DA and DC groups are clustered, respectively, indicating that the expression patterns of metabolites in the sows at diestrus and metestrus are different. The samples in the same group may have similar functions or participate in the same biological process (Figure 9). Two benzothiophene and L-(+)-valine, ethyl mercury ions were extremely significant positive correlation (*P* < 0.01), aminomethyl phosphonic acid and ethyl mercury ions were significantly negative correlation (*P* < 0.05), sucrose and 6-hydroxy-1,2-adipic acid dioctyl ester, misha ossification alcohol, 2-ammonia-4-oxygen generation butyrate were extremely significant positive correlation (*P* < 0.01), and (3R, 4S, 9R, 11R)-27-(4-hydroxy phenyl)-4-methyl-3,9,11-seven alkoxy three alcohol has significant negative correlation (*P* < 0.05; Tables 2–4).

**TABLE 2:** The significant different metabolites of positive and negative ion modes (top 10).

ID	Metabolites	Model	FC	*P*	VIP	Up/Down
Com_8790_pos (P1)	Dibenzothiophene	+	2.52	<0.01	2.01	Up
Com_3958_pos (P2)	2-Acetamido-2-deoxy-alpha-D-galactopyranosyl-(1->3)-[6-deoxy-alpha-L-galactopyranosyl-(1->2)]-beta-D-galactopyranosyl-(1->4)-D-glucose	+	0.3	<0.01	2.33	Down
Com_8630_pos (P3)	Ceanothine B	+	0.39	0.01	1.92	Down
Com_8467_pos (P4)	3,3′,3′′,3′′′-[3,8,13,17-Tetrakis(carboxymethyl)-8,13-dimethyl-8,12,13,18,20,23-hexahydro-1H,7H-porphine-2,7,12,18-tetrayl]tetrapropanoic acid	+	2.64	0.01	2.26	Up
Com_6980_pos (P5)	spiro[3H-indole-3,5′(4′H)-thiazol]-2-ol, 2′-(methylthio)-	+	0.42	0.01	2.31	Down
Com_13726_pos (P6)	L-(+)-Valine	+	2.64	0.01	1.93	Up
Com_6320_pos (P7)	Ethyl mercury ion	+	8.39	0.02	4.18	Up
Com_3964_pos (P8)	Aminomethylphosphonic acid	+	0.36	0.02	2.75	Down
Com_3720_pos (P9)	2-Amino-9-(2-deoxypentofuranosyl)-3,9-dihydro-6H-purin-6-one	+	2.62	0.02	2.31	Up
Com_4939_pos (P10)	*N*-(1-{[methyl(2-methyl-2-propanyl)carbamoyl]amino}ethyl)-alpha-asparagine	+	0.29	0.02	4.28	Down
Com_1306_neg (N1)	6-Hydroxy-1,2-hexanediyl dioctanoate	−	0.43	<0.01	1.67	Down
Com_4818_neg (N2)	Molindone	−	0.34	<0.01	2.41	Down
Com_1461_neg (N3)	Maxacalcitol	−	0.35	<0.01	2.05	Down
Com_3334_neg (N4)	2-Ammonio-4-oxobutanoate	−	0.45	<0.01	1.52	Down
Com_986_neg (N5)	(3R,4S,9R,11R)-27-(4-Hydroxyphenyl)-4-methyl-3,9,11-heptacosanetriol	−	4.76	<0.01	3.64	Up
Com_2165_neg (N6)	(R)-3-Hydroxy myristic acid	−	0.48	<0.01	1.42	Down
Com_2393_neg (N7)	Methylethylmethane	−	0.35	0.01	1.96	Down
Com_2977_neg (N8)	Manoalide	−	0.49	0.01	1.33	Down
Com_5521_neg (N9)	Sucrose	−	0.28	0.01	2.34	Down
Com_5715_neg (N10)	(2S,4R,9a′S)-1′-Hydroxy-4-{2-[(1S)-1-hydroxyethyl]-4-oxo-3(4H)-quinazolinyl}-2′,2′-dimethyl-1′,9a′-dihydro-3H-spiro[furan-2,9′-imidazo[1,2-a]indole]-3′,5(2′H,4H)-dione	−	3.34	0.01	2.33	Up

**TABLE 3:** The correlation of significant different metabolites of the top 10 in positive ion mode (top 10).

Metabolites	P1	P2	P3	P4	P5	P6	P7	P8	P9	P10
P1	1									
P2	−0.444	1								
P3	−0.459	0.970**	1							
P4	0.410	−0.435	−0.589	1						
P5	−0.783**	0.406	0.448	−0.437	1					
P6	0.789**	−0.236	−0.237	−0.014	−0.613*	1				
P7	0.971**	−0.374	−0.381	0.350	−0.783**	0.755**	1			
P8	−0.644*	0.318	0.364	−0.487	0.863**	−0.556	−0.626*	1		
P9	0.331	−0.523	−0.498	0.211	−0.422	0.106	0.329	−0.211	1	
P10	−0.770**	0.333	0.394	−0.459	0.872**	−0.500	−0.768**	0.640*	−0.486	1

*Difference is significant.

**Difference is extremely significant.

**TABLE 4:** The correlation of significant different metabolites of the top 10 in negative ion mode (top 10).

Metabolites	N1	N2	N3	N4	N5	N6	N7	N8	N9	N10
N1	1									
N2	0.648*	1								
N3	0.959**	0.555	1							
N4	0.842**	0.549	0.902**	1						
N5	−0.639*	−0.635*	−0.614*	−0.683*	1					
N6	0.699*	0.766**	0.725*	0.820**	−0.717*	1				
N7	0.480	0.621*	0.491	0.774**	−0.677*	0.836**	1			
N8	0.579	0.246	0.567	0.770**	−0.497	0.501	0.656*	1		
N9	0.817**	0.723*	0.879**	0.888**	−0.658*	0.895**	0.734*	0.434	1	
N10	−0.619*	−0.635*	−0.554	−0.521	0.120	−0.477	−0.354	−0.422	−0.502	1

*Difference is significant.

**Difference is extremely significant.

**FIGURE 10:**
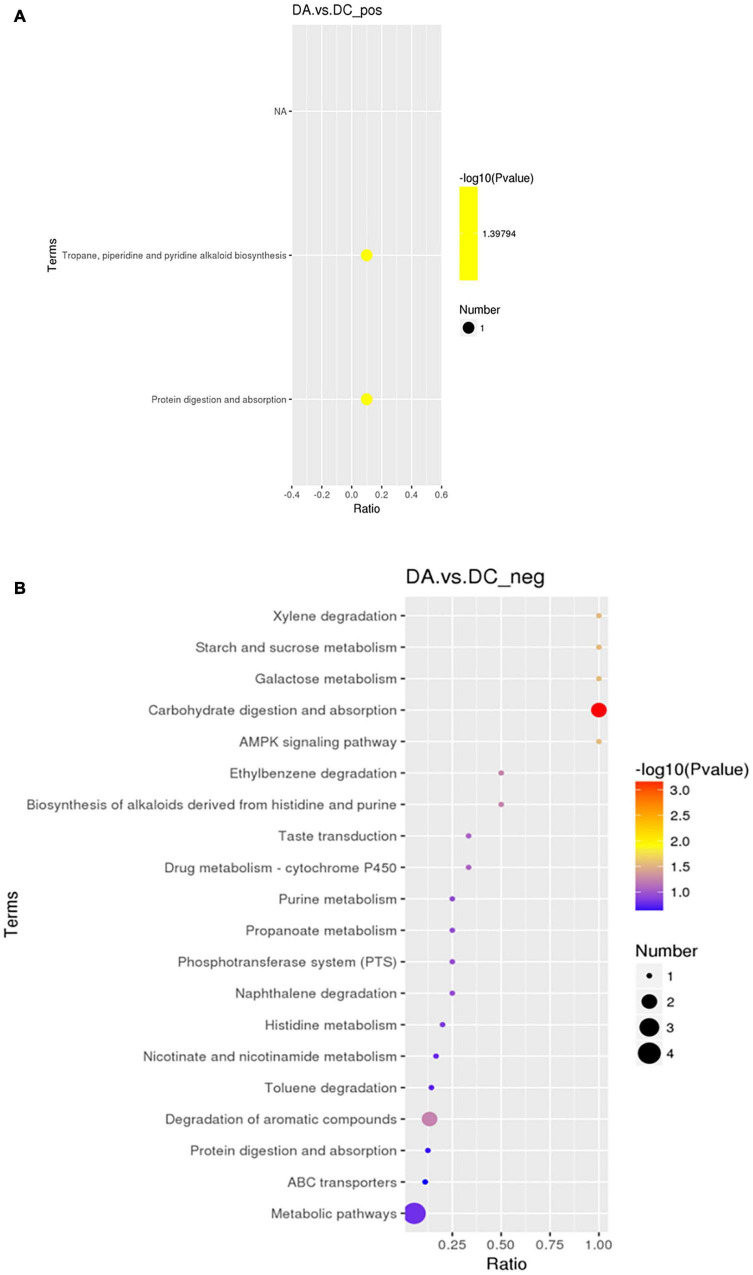
The KEGG enrichment map. **(A)** Positive ion mode and **(B)** negative ion mode.

The KEGG analysis (Figure 10) showed that in the positive ion mode, the significant differences were in the biosynthesis pathways of tolane, piperidine, and pyridine alkaloids involved by piperidine (*P* < 0.05) and protein digestion and absorption pathways (*P* < 0.05). In the negative ion mode, propionic acid, benzyl butyrate, and sucrose are involved in carbohydrate digestion and absorption of pathways (*P* < 0.01). Sucrose is involved in galactose metabolism pathways (*P* < 0.05), and starch and sucrose metabolism pathways (*P* < 0.05). 4-methylcatechol participated in dimethyl benzene degradation pathways (*P* < 0.05) and 5-aminoimidazole-4-formamide nucleotide (AICAR) was involved in the AMPK signaling pathway (*P* < 0.05; Table 5).

**TABLE 5:** The significant difference of the Kyoto Encyclopedia of Genes and Genomes (KEGG) enrichment table of the positive and negative ion modes.

Pathway	*P* value	Mode	Differential metabolites involved in this pathway
Tropane, piperidine, and pyridine alkaloid biosynthesis	0.04	+	Piperidine
Protein digestion and absorption	0.04	+	Piperidine
Carbohydrate digestion and absorption	<0.01	−	Benzyl butyrate, propionic acid, and sucrose
Galactose metabolism	0.03	−	Sucrose
Starch and sucrose metabolism	0.03	−	Sucrose
Xylene degradation	0.03	−	4-methyl catechol
AMPK signaling pathway	0.03	−	AICAR

### Analysis of Correlations Between Different Microbiota and Metabolites

**FIGURE 11:**
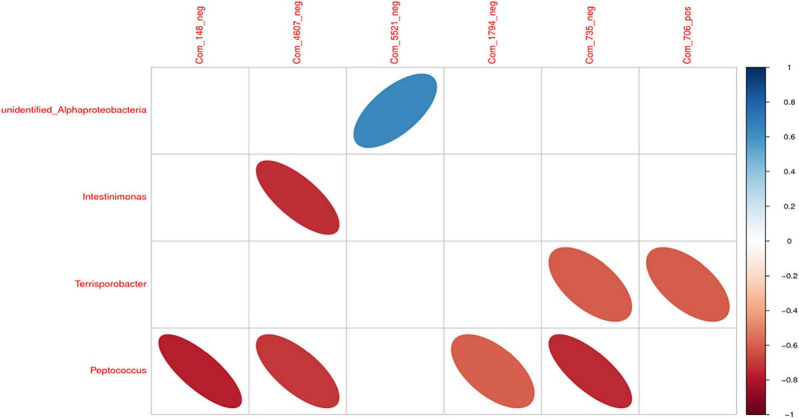
The correlation heat map of differential intestinal microbiota and metabolites. Note: Com_148_neg: propionic acid; Com_4607_neg: benzyl butyrate; Com_5521_neg: sucrose; Com_1794_neg: AICAR; Com_735_neg: 4-methyl catechol; Com_706_pos: piperidine.

**FIGURE 12:**
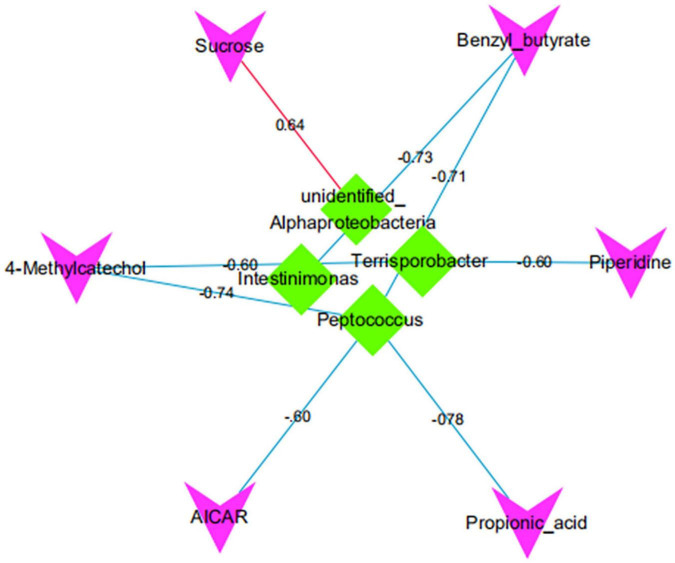
The correlation network map of differential intestinal microbiota and metabolites. Red/green indicates that the shape represents metabolite/bacteria, red/blue lines represent positive/negative correlation, and numbers represent correlation coefficients.

There was a significant positive correlation between *unidentified_Alphaproteobacteria* and sucrose (*P* < 0.05). However, *Intestinimonas* with benzyl butyrate exhibited a significantly negative correlation (*P* < 0.05). There was a significant negative correlation between *Peptococcus* and propionic acid, benzyl butyrate, AICAR, and 4-methyl catechol (*P* < 0.05; Figures 11, 12).

## Discussion

Animal intestines are home to many complex and diverse microbiota, and there is a mutually beneficial symbiosis between the microbiota and the host (Thompson and Trexler, 1971; Hayakawa et al., 2016). Recently, it has been demonstrated that the gut microbiota is a critical component of host physiology (Sekirov et al., 2010). Considering the important, dynamic, and reflective role of gut microbiota in host physiology and metabolism, conducting a study on estrus’s effect on gut microbes is optimizing sow reproductive performance and production management (Hooper et al., 2002; Pluznick, 2014). However, little information has been conducted on the gut microbiota in Diannan small ear sows. Therefore, we explored the possible association between estrus and gut microbiota and microbial metabolites using high-throughput 16S rRNA sequencing and LC-MS non-targeted metabolome technology at diestrus and metestrus.

Our findings revealed that *Firmicutes* and *Bacteroidetes* are the most abundant phylum in sow’s intestines, accounting for more than 90% of the total sequence, which is consistent with an earlier study (Kim et al., 2012; Poole et al., 2013). *Firmicutes* and *Bacteroidetes* play a critical role in maintaining the normal physiological function of the intestinal tract and microbial balance, which is of great significance to the growth and health of animals (Armougom and Raoult, 2008; Xu et al., 2019).

This study showed significant differences in the microbial structure were identified in sows at diestrus and metestrus, and the abundance of *Ruminococcus_sp_YE281*, *Treponema_berlinense*, and *unidentified_Alphaproteobacteria* in the feces of Diannan small ear sows at metestrus were significantly higher than those at diestrus. *Ruminococcus_sp_YE281* is a species of bacteria under the *Ruminococcus*. *Ruminococcus* can degrade cellulose in plant cell walls (Fernando et al., 2010; Guan et al., 2018). *Treponema_berlinense* is a genus of bacteria under the *Treponema*. *Treponema* can degrade oligosaccharides, polysaccharides, lignocellulose, and indigestible substances (Guo, 2008). *unidentified_Alphaproteobacteria* is a genus of bacteria under the *Proteobacteria*. Shin et al.’s (2015) study have shown that *Proteobacteria* contains a variety of pathogenic bacteria and are a marker of instability in the intestinal microbiota. However, some studies have also shown that *Proteobacteria* can assist in the degradation of lignin (Fang et al., 2012). Whether the high abundance of unidentified_Alphaproteobacteria is beneficial or harmful to sows at metestrus needs further verification. It is worth noting that Spirobacteriaceae was found in the feces of sows in metestrus. Spirobacteriaceae and some species have been identified in humans as the leading cause of syphilis, Lyme disease, recurrent fever, and other diseases (Karami et al., 2014). There may be due to that sows transition from restlessness to arching and standing still, dull eyes, anorexia in metestrus (Tang et al., 2018).

Function analysis indicated that more bacterial genes are involved in carbohydrate metabolism, amino acid metabolism, energy metabolism; that is correlated with the enrichment of *Bacteroidetes* in the gut of Diannan small ear sows. *Bacteroidetes* are good at degrading non-fibrous substances and are the primary polysaccharide degradation and utilization bacteria, which can improve the utilization rate of carbohydrates, proteins, and other substances by the host and enhance the immunity of the host (Becker et al., 2014).

The metabolomics results of this study found that the propionic acid, benzyl butyrate, and sucrose content at metestrus Diannan small ear sows’ feces was significantly higher than at diestrus, these metabolites are involved in carbohydrate digestion and absorption pathway, which is consistent with the previous study results (Sankar and Archunan, 2008; Flores et al., 2012). We also found an extremely significant increase in piperidine concentration at metestrus sows feces than at diestrus, which involved the protein digestion and absorption pathways. At metestrus, sows showed anorexia, and the continuous excitement and agitation led to the reduction of blood glucose levels (Jezková and Smrcková, 1990). Therefore, to balance the blood glucose level, and maintain the nutritional needs of the body Diannan small ear sow needs to digest and absorb more carbohydrates and protein.

During estrus, the sows secrete a large number of sex hormones such as luteinizing hormone and follicle-stimulating hormone, while the concentration of progesterone gradually decreases (Toyama et al., 2018). Our study indicated that the concentration of AICAR in estrus sow’s feces was significantly higher in metestrus than in diestrus. AICAR is an activator of AMPK, which is involved in the AMPK signaling pathway. Tosca et al. (2005) found that *in vitro* culture of mouse granulosa cells, activation of AMPK by AICAR significantly reduced progesterone secretion by affecting gonadotropin secretion. Kimura’s earlier studies found that animal feces contained compounds associated with estrus, especially in females (Kimura, 2001). Sankar and Archunan confirmed that changes in compounds in feces could be an effective method to detect estrus in animals (Sankar and Archunan, 2008). Feces are one of the main agents for the elimination of metabolites in animals, so it may potentially convey a great deal of information about also animals internal physiology and provide a source of chemical signals. Therefore, this study suggested that AICAR may be a potential marker of estrus sows’ feces.

The correlation between intestinal microbiota and metabolites suggests that intestinal microbiota plays an essential role in maintaining body health (Chow et al., 2010). Our study found that the *unidentified_Alphaproteobacteria* was significantly positively correlated with sucrose, indicating that *unidentified_Alphaproteobacteria* can improve the carbohydrate digestion and absorption pathway of Diannan small ear sows at metestrus. It proves that unidentified_Alphaproteobacteria is a beneficial bacteria for Diannan smaller sows at metestrus. Thingholm et al. (2019) found that *Intestinimona* was significantly associated with obesity, and the abundance of *Intestinimonas* was significantly reduced when people were obese. In this study, *Intestinimonas* and benzyl butyrate, significantly have a negative correlation. These results show that when the Diannan smaller sows are at metestrus, the host’s carbohydrate digestion and absorption pathway can be improved by reducing the abundance of intestinimonas to meet the nutritional needs of Diannan smaller sows at estrus and maintain the weight of Diannan smaller sows at estrus. In addition, we also discovered that *Terrisporobacter* and 4-methyl catechol, piperidine, *Peptococcus* and, propionic acid, benzyl butyrate, AICAR, 4-methyl catechol significantly have a negative correlation, but the specific interaction mechanism still needs to be explored.

## Data Availability Statement

The data presented in the study are deposited in the GenBank repository, accession numbers ON026865-ON028634.

## Ethics Statement

The animal study was reviewed and approved by Institutional Animal Care and Use Committee of Yunnan Agricultural University. Written informed consent was obtained from the owners for the participation of their animals in this study.

## Author Contributions

SMZ and XCG were responsible for the conception and design of the experiment and participated in drafting the manuscript. JHZ, HCS, XQZ, MHY, YH, HBP, and YGZ participated in the acquisition of the data and analyses of the results and agreed to be accountable for the accuracy and integrity of the data. All authors read and approved the final manuscript.

## Conflict of Interest

The authors declare that the research was conducted in the absence of any commercial or financial relationships that could be construed as a potential conflict of interest.

## Publisher’s Note

All claims expressed in this article are solely those of the authors and do not necessarily represent those of their affiliated organizations, or those of the publisher, the editors and the reviewers. Any product that may be evaluated in this article, or claim that may be made by its manufacturer, is not guaranteed or endorsed by the publisher.

## Footnotes

1
http://cutadapt.readthedocs.io/en/stable/

2
http://www.drive5.com/uparse/
